# Characteristics of adult retroperitoneal lymphangioma: a single center Chinese cohort study of 15 cases

**DOI:** 10.1007/s12672-024-01143-5

**Published:** 2024-07-04

**Authors:** Jie Liu, Zhi-Han Zhong, Shu-Sen Zheng

**Affiliations:** 1 Division of Hepatobiliary and Pancreatic Surgery, Department of General Surgery, The First Affiliated Hospital, Zhejiang University School of Medicine, Hangzhou, 310003 Zhejiang China; 2 General Surgery, Cancer Center, Department of Hepatobiliary & Pancreatic Surgery and Minimally Invasive Surgery, Zhejiang Provincial People’s Hospital, Affiliated People’s Hospital, Hangzhou Medical College, Hangzhou, 310003 Zhejiang China

**Keywords:** Diagnostic, D2-40, CD34, Prognosis, Retroperitoneal lymphangioma

## Abstract

**Background:**

Lymphangioma is a rare cystic tumor that occurs across different locations. Retroperitoneal lymphangioma accounts for about 1% of all lymphangiomas. In this study, we report the clinicopathological features of retroperitoneal lymphangioma and describe our experience in treating this disease.

**Methods:**

We collected clinical data from all patients who were pathologically diagnosed with retroperitoneal lymphangioma at Zhejiang Provincial People's Hospital, between June 2013 and August 2022.

**Results:**

The 7 and 8 male and female patients analyzed herein had a mean age of 48.6 (SD 14.24) years at diagnosis. The mean duration of follow-up was 4.7 years. Among them, 66.67% were asymptomatic, with the rest manifesting abdominal pain, nausea, low back pain and elevated blood pressure as the main symptoms. Preoperative diagnosis and evaluation of cysts were mainly performed via computed tomography (CT) (n = 10, 66.67%) or magnetic resonance imaging (MRI) (n = 8, 53.33%). All patients were completely resected following surgery. Immunohistochemical analysis, performed on 6 patients, revealed that they were positive for D2-40. A total of 4, 4 and 3 patients were positive for CD31, CD34 and SMA, respectively. Moreover, the study cohort had an average postoperative hospital stay of 6.6 days. Follow up, after the end of the study, revealed no relapse in any of the 15 patients.

**Conclusions:**

Lymphangioma is a benign tumor of the lymphatic system. Although typical imaging features can be accurate for preoperative diagnosis, histological examination is crucial to final confirmation. Complete surgical resection is the best option to limit the risk of recurrence in cases with symptomatic lesions.

## Introduction

Lymphangioma is a rare benign tumor that often occurs in children, with about 90% of all cases diagnosed before the age of 2 years. Prone sites of lymphangioma include the neck, head, and axillary regions. Notably, lymphangioma located in retroperitoneal is rare, and it is commonly caused by dysplasia of retroperitoneal lymphatic vessels or their traumatic rupture [1, 2]. The tumor is commonly confused with other retroperitoneal cystic tumors, including cystic tumors caused by the kidney and pancreas [3]. Depending on the location, it may be misdiagnosed as pancreatic pseudocyst, ovarian cyst, renal cyst or other diseases [4].

Patients with retroperitoneal lymphangioma usually lack specific symptoms, and most of them are found by accident. Although some patients may suffer from abdominal pain, abdominal distension, lumbar pain and various forms of discomfort, others may exhibit symptoms associated with mass complications, such as bleeding, infection, cyst rupture or compression of adjacent organs [5]. Preoperative diagnosis is commonly achieved via computer tomography (CT) and magnetic resonance imaging (MRI), while final diagnosis requires surgical exploration and histopathological results. Studies demonstrated that incomplete resection is the only reason of recurrence, and complete resection is therefore commonly recommended as the gold standard treatment [6, 7]. In this study, we analyzed clinical and imaging features, as well as immunohistochemical and pathological findings of 15 patients with retroperitoneal lymphangioma. We further conducted a follow-up to confirm the benign nature of the tumor.

## Materials and methods

We retrospectively enrolled 15 patients with retroperitoneal lymphangioma, who were confirmed by pathology from June 2013 to August 2022, at Zhejiang provincial people’s hospital. Each patient voluntarily provided a written informed consent, and the study was approved by the ethics committee of Zhejiang provincial people's hospital. We also retrospectively collected detailed demographic and clinical data, then performed pathological and immunohistochemical assays via hematoxylin and eosin staining, avidin biotin complex immunoperoxidase technique. Finally, we followed up the long-term prognosis of patients via telephone.

## Results

All patients included in this study, comprising 7 males and 8 females (male ratio of about 1:1) were aged between 30 and 76 years (48.6 ± 14.24 years). Details on their clinical data are outlined in Table 1. Most of the patients (n = 10, 66.67%) were asymptomatic and were diagnosed by regular physical examination. The rest (5 patients) manifested the following symptoms; pain and discomfort in the back and waist (n = 2), abdominal pain (n = 1), nausea (n = 1), and increased blood pressure with dizziness and weight loss (n = 1). No mass can be touched from body surface in all patients, and there was no elevation of tumor markers. For treatment, all patients underwent complete surgical resection, including laparoscopic resection (n = 14) and robot assisted resection (n = 1). All 15 patients were followed up via telephone, for an average follow-up time 4.7 (median 4) years. We found neither recurrence nor complaints of discomfort during follow-up.

**Table 1:** Clinicopathological summary of 15cases of retroperitoneal lymphangioma

Variables	Case, N (%)	Mean	Standard deviation
Age at diagnosis, year		46.8	14.24
≤ 60	11 (73.33%)		
> 60	4 (26.67%)		
Gender			
Male	7 (46.67%)		
Female	8 (53.33%)		
Clinical presentation			
Increased blood pressure and weight loss	1 (6.67%)		
Abdominal pain	1 (6.67%)		
Waist and back pain	2 (13.33%)		
Nausea	1 (6.67%)		
No symptoms	10 (66.67%)		
Outcome			
Rechecked regularly and had no recurrence or metastasis	15(100%)		
Recrudescence	0 (0%)		
Tumor size(mm)		76.53	36.54
> 30, ≤ 50	5 (33.33%)		
> 50, ≤ 70	3 (20.00%)		
> 70, ≤ 90	2 (13.33%)		
> 90, ≤ 110	1 (6.67%)		
> 110	4 (26.67%)		
Tumor classification			
Cystic lymphangioma	12 (80.00%)		
Cavernous lymphangioma	3 (20.00%)		
Capillary lymphangioma	0 (0.00%)		
Single room/multi room			
Single room	7 (46.67%)		
Multi room	8 (53.33%)		
CT enhancement pattern of mass			
No enhancement	9 (60.00%)		
Moderate enhancement	1 (6.67%)		
Lack	5 (33.33%)		
Tumor location			
Left retroperitoneal	10 (66.67%)		
Right retroperitoneal	5 (33.33%)		
Postoperative hospitalization days		6.6	7.78
2–4	11 (73.33%)		
6	1 (6.67%)		
9	1 (6.67%)		
21	1 (6.67%)		
30	1 (6.67%)		

### Imaging results

**Fig. 1:**
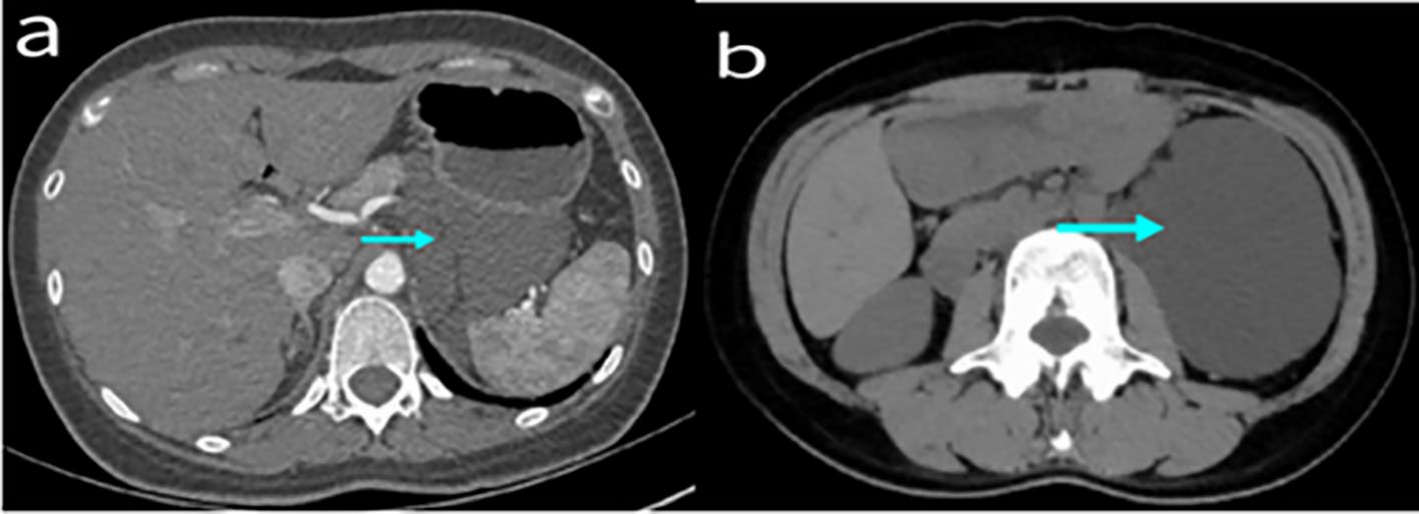
Figure **a** shows Enhanced CT images of retroperitoneal lymphangioma; Figure **b** shows CT scan without contrast

**Fig. 2:**
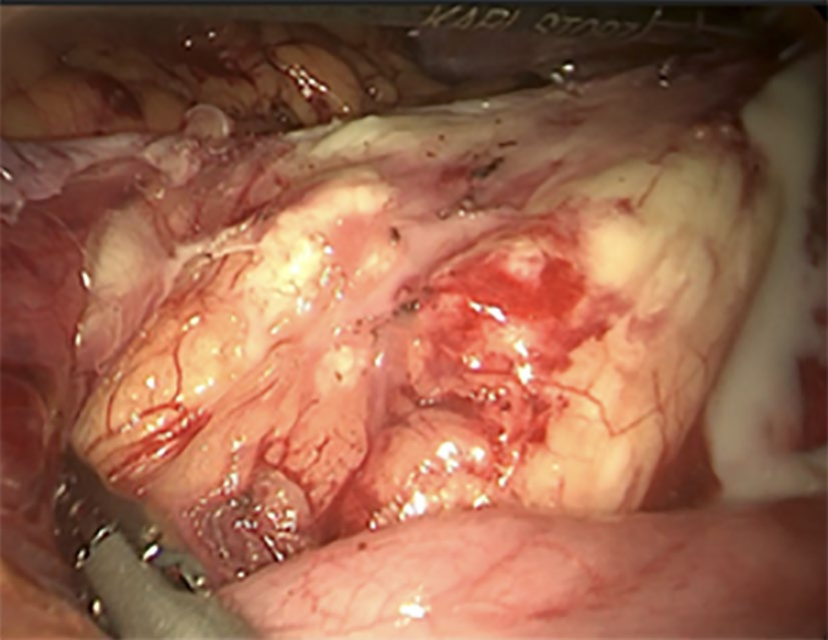
Retroperitoneal cystic lymphangioma with white emulsion leakage during operation

**Fig. 3:**
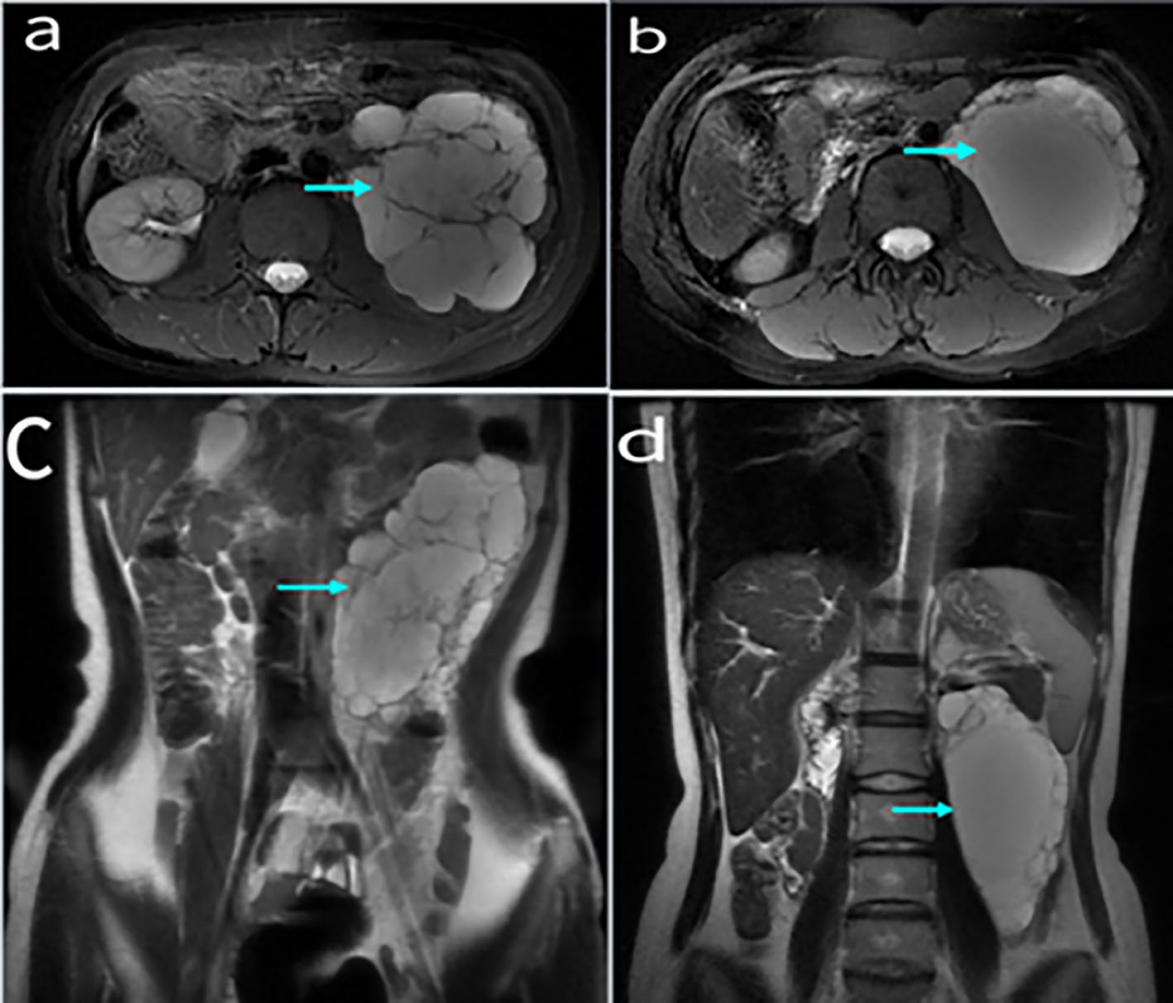
Enhanced MRI image of retroperitoneal lymphangioma

A total of 10 patients had enhanced CT data (Fig. 1), with a clear tumor boundary. Among them, 9 patients did not exhibit obvious enhancement, with most of them appearing as a yellow clear liquid or clear liquid during operation. One case was moderately enhanced, and was subsequently diagnosed with a multilocular retroperitoneal cystic lymphangioma. White milky liquid was seen during the operation (Fig. 2), and fat capsule wall like tissue was contained. Enhanced MRI data was obtained in 8 patients (Fig. 3), of which 6 exhibited low and high signals on T1WI and T2WI, respectively. 2 patients had long signals on both T1WI and T2WI. 3 cases exhibited enhancement of the capsule wall and 1 of which exhibited enhancement of internal compartment as well, and 5 cases showed no enhancement. Preoperative imaging diagnosis revealed that 13 cases (86.67%) were lymphangioma, 1 case was considered as stromal tumor and neurogenic, and 1 case was considered as cystic space occupying lesion.

### Gross pathology

Among the 15 patients with retroperitoneal lymphangiomas, 12 patients were diagnosed with cystic and 3 patients were diagnosed with cavernous lymphangiomas. Notably, 10 cases were located in the left retroperitoneum as follows; 3 behind the splenogastric space, 3 around the left kidney, 2 behind the tail of the pancreatic body, 1 in the horizontal segment of the duodenum, and 1 behind the descending colon. On the other hand, 5 cases were located in the right retroperitoneum, including 3 and 2 cases around the right kidney and behind the hepatic flexure of the colon, respectively. The average diameter of masses was 76.53 mm ± 36.54 (range 31 to 140 mm). All tumors had clear boundaries, of which 8 with unilocular boundaries and 7 with multilocular boundaries.

### Immunohistochemical findings

**Fig. 4:**
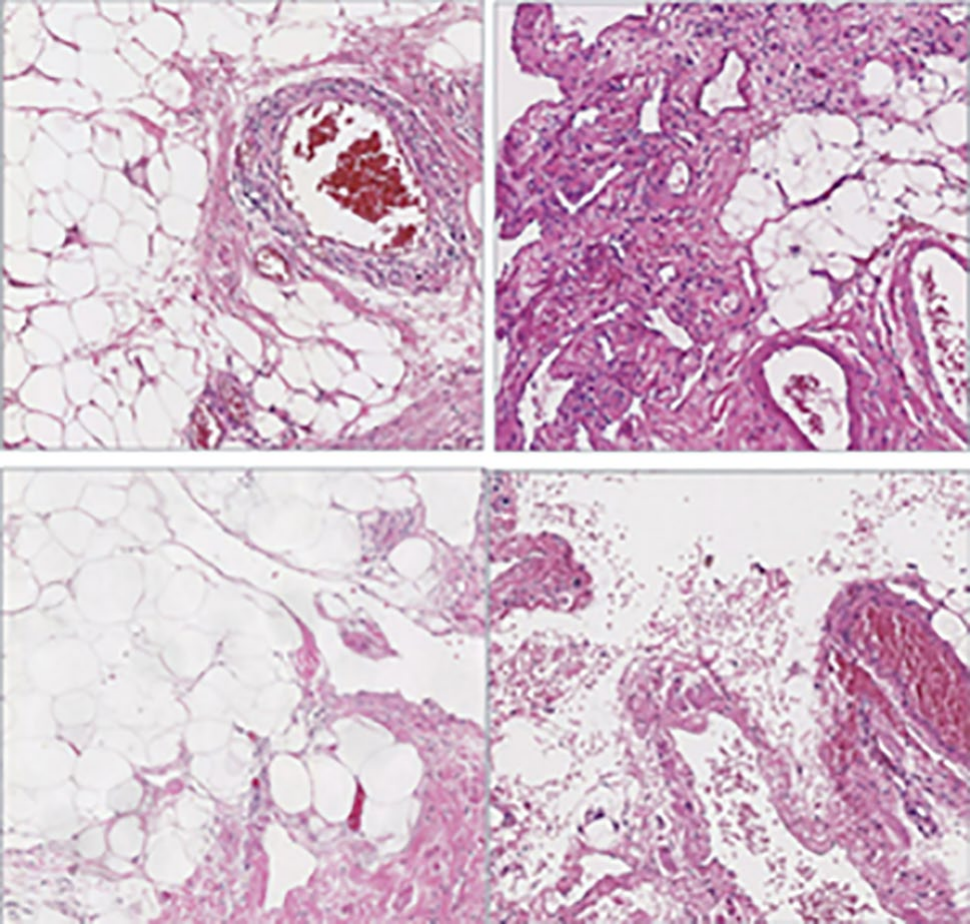
HE piceure of four cases of retroperitoneal lymphangioma. (All pictures have 10x10 magnification)

**Fig. 5:**
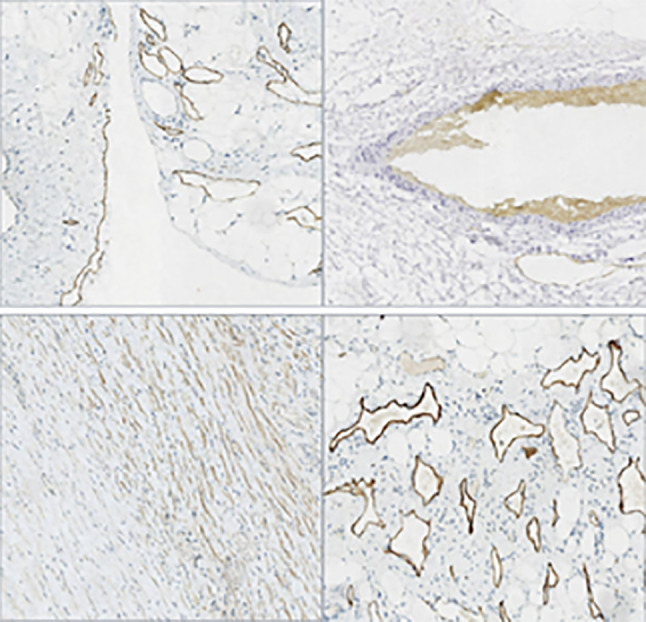
Four cases of retroperitoneal lymphangioma were D2-40 positive (All pictures have 10x10 magnification)

**Fig. 6:**
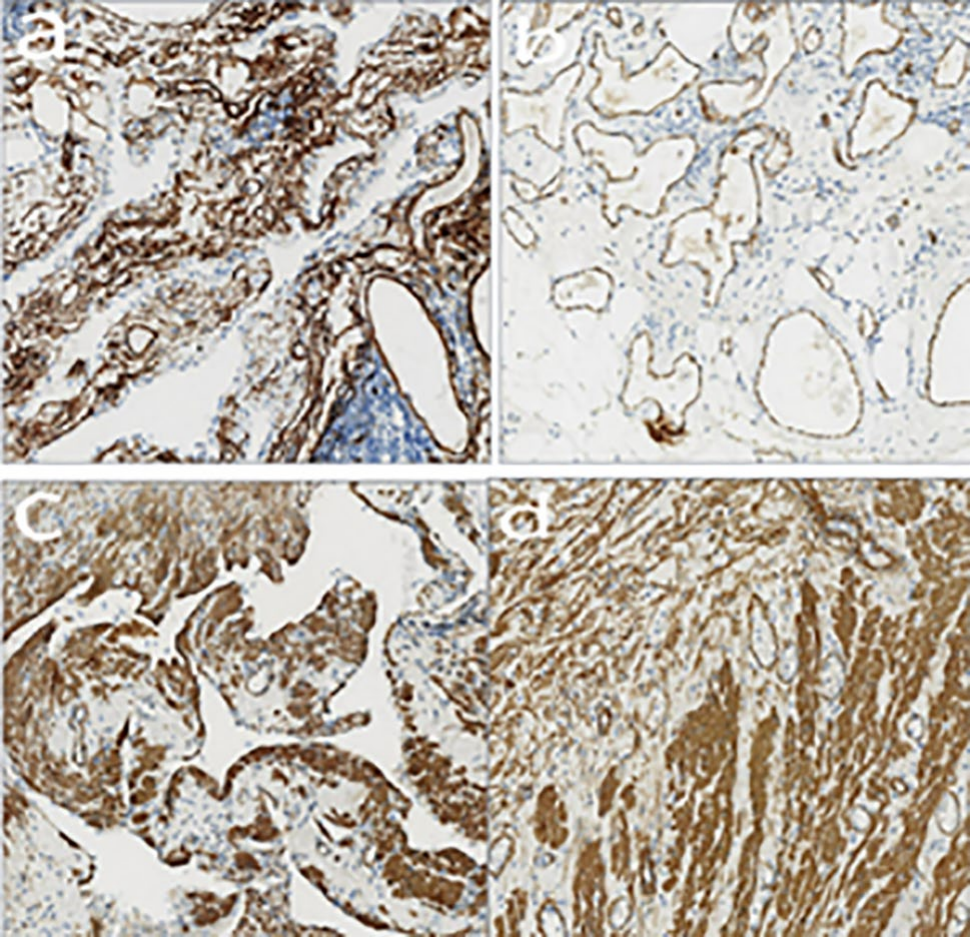
Figure **a** shows CD34 positive; Figure **b** shows CD31 positive; Figure **c** and **d** shows SMA positive. (All pictures have 10x10 magnification)

Results of immunohistochemical staining, performed on specimens from all 15 patients, are shown in Fig. 4. Summarily, the specimens revealed dilated lymphatic vessels lined with endothelial cells and abundant lymphoid tissues. D2-40 was detected in 6 patients (Fig. 5), while 4, 4 and 3 cases were positive for CD31, CD34 and SMA, respectively (Fig. 6). CK5/6 and calretinin were negative.

## Discussion

We report occurrence of retroperitoneal lymphangioma, a rare benign tumor that is mainly seen on children [8, 9]. To our knowledge, this is the largest single center cohort of adult retroperitoneal lymphangiomas reported to date. Studies in children have revealed a similar sex ratio, although in some instances boys may be slightly affected than girl [10]. The current study based on an all-adult cohort revealed an almost similar ratio of male to female patients, which was consistent with previous findings from children’s groups. Previous studies have shown that the tumor mass was always located on one side [11, 12]. Saadi et al. reviewed five patients with retroperitoneal lymphangioma, and found that three had the tumor on the right side, one manifested it on the left side, while one had it in the pelvis [11]. In the present study, 10 cases exhibited the tumor in the left retroperitoneum while 5 cases manifested it in the right side, which was contrary to the previous study. Most retroperitoneal lymphangiomas are not only asymptomatic, but also found by accident [13]. The most common clinical manifestations include abdominal pain and nausea, which are mostly caused by growing masses. In the present study, we compared the tumor to abdominal lymphangioma, owing to a lack of retroperitoneal lymphangioma cohort. Xiao et al. reviewed 12 cases of adults with abdominal lymphangioma, and found that half of them were asymptomatic and could be occasionally detected via physical examination [4]. Most of the patients in the present study were asymptomatic, which was consistent with previous studies. Apart from abdominal pain and nausea, we also observed some special symptoms, which may be attributed to the different locations of the masses. For instance, one patient with mass locating behind the left hilum of kidney, exhibited elevated blood pressure (with a high of 190/100mmhg), dizziness and weight loss. Although the largest mass in this study directly reached 140 mm, no mass could be touched in all patients during physical examination. Studies have shown that enlargement of masses may elicit occurrence of symptoms such as mass rupture, bleeding, infection, and even intestinal obstruction or volvulus [10]. This necessitatesemergency surgical treatments.

For the lack of specific clinical signs, preoperative imaging examination will guide the diagnosis. Ultrasound is considered as the first-line examination method and a suitable tool for disease screening. Narrow and clear borders, coupled with strong echo, and low echo fluid in the center as well as no blood flow signal in color Doppler flow imaging represent the typical lymphangioma characteristic [4]. In the present study, we employed CT and MRI scans for effective auxiliary examinations. Notably, CT provides a better platform for visualization of lesion location and mass size, as well as the relationship with other assessments and other surrounding organs compared to ultrasound. Typically, CT allows visualization of a tumor with uniform content, and low density, without obvious enhancement [11]. The CT value of lymphangioma mass is between those of water and fat, which is consistent with our study. Although many publications indicate that retroperitoneal lymphangioma does not calcified [14]. Davidson et al. included two lymphangioma cases with calcification in their research queue [15]. Our study also found a case of lymphangioma with calcification within the mass through CT scan. As for MRI, previous studies have shown that most lesions are fluid filled masses with low T1 and high T2 signal intensities, respectively [16]. Previous studies have shown that preoperative diagnosis of lymphangioma is rare [4, 17]. However, in the present study, imaging results indicated that 13 patients (86.67%) had lymphangioma, 1 case was diagnosed with cystic disease and another 1 case was misdiagnosed as stromal tumor (neurogenic consideration). Notably, the misdiagnosed tumor location was closely associated with the horizontal segment of the duodenum. We also found evidence of bleeding inside the tumor, which affected efficiency of imaging-based diagnosis.

Complete resection remains the best method for treatment of this disease. In the present study, all the patients underwent complete resection, and none of them relapsed after an average follow-up of 4.7 years. Although active puncture decompression can be performed for oversized cysts during operation, care should be taken to avoid emptying of the cyst fluid. This ensures that the cyst wall maintains a certain tension and facilitates complete dissection. The wall of cyst is often thin and easy to rupture, thus it should be handled carefully during operation. In some cases, the cyst wall was broken during the operation, resulting in its collapse that makes the operation difficult. In such a case, normal saline should be injected into the cyst after closing the wound, to make it regain tension and facilitate peeling off the cyst wall. In cases that cannot be completely resected, the capsule should be resected as much as possible, after which the residual tissue should be smeared with 3–5% iodine tincture and cauterized using an electric knife. In the case of peripheral tissue involvement, resection should also include resection of peripheral tissue to ensure complete resection of the tumor. This is because incomplete resection may exacerbate the risk of recurrence (50% compared to 7% observed in cases with complete resection) [18–20].

Fujishiro et al. [21] reported a case of extensive adhesion between retroperitoneal lymphangioma and duodenum and pancreatic head, and performed pancreatoduodenectomy. One patient in the current study underwent partial resection of duodenum as the mass was located in the horizontal segment of the duodenum. The average length of hospitalization of all patients after operation was 6.6 days. Moreover, one patient had postoperative chyloid ascites and lymphatic leakage. To avoid this, clinicians need to ensure they completely remove small ducts during operation.

Histologically, lymphangiomas are characterized by 3 distinguishable subtypes, namely capillary, spongy and cystic, of which the latter is the most commonly occurring. Most of the lymphangiomas in the present study were the cystic subtypes, with only 3 found to be cavernous lymphangiomas. At present, the disease is mainly caused by congenital and acquired factors. Between them, congenital factors refer to development of fetal lymphatic vessels in the embryonic period that result in blind ends and misconfiguration of terminal lymphatic vessels, thereby causing abnormal lymph reflux and formation of cystic expansion [22].Acquired factors include secondary infection, inflammation, trauma, surgery, and other injuries that cause abnormal lymphatic reflux and formation of cystic masses [23].

In the present study, we employed pathological and immunohistochemical analyses to finally confirm the disease. Pathologically, lymphangioma typically manifests as local deposition and small lymphatic lacunae that can be seen in the lymphoid tissue of the capsule wall. The wall is thin, with evidence of endothelial cells, and there is generally no cuboidal epithelium or columnar epithelium. It is also covered with glia and smooth muscle fibers, and lymphocyte aggregation can be seen in the stroma [24]. Ellis et al. reported that lymphangioma patients are also positive for D2-40, Prox1 and CD31, but negative for CD34 and ckae1/AE3 staining [25]. However, Gui et al. used immunohistochemistry to reveal endothelial cell expression factor VIII related antigens CD31 and CD34 in cystic lymphangioma [26]. Immunohistochemistry results in the present study revealed that 6 patients were positive for D2-40, 4 were positive for CD31, with an additional 4 positive for CD34. This indicated that lymphangioma can induce expression of markers associated with capillaries and lymphatic endothelium.

## Conclusion

In summary, we report 15 cases of retroperitoneal lymphangioma, a rare benign disease whose initial symptoms are hidden, which is the largest single center cohort of adults. Preoperative CT and MRI examination can be used to make a definite diagnosis, and a few tumors will have calcification inside. In cases where the tumor is closely related to surrounding organs and there is internal bleeding, certain difficulties should be expected during preoperative diagnosis. Complete resection of the tumor remains the main mode of treatment. In cases where the tumor is mainly adhered to surrounding organs, removal of some of these organs may be necessary. Histological examination is imperative to final diagnosis.

## Data Availability

The datasets used and/or analyzed during the current study available from the corresponding author on reasonable request.

## Code availability

Contact with the first author if necessary.
